# Characterization of Sin1 Isoforms Reveals an mTOR-Dependent and Independent Function of Sin1γ

**DOI:** 10.1371/journal.pone.0135017

**Published:** 2015-08-11

**Authors:** Yuanyang Yuan, Bangfen Pan, Haipeng Sun, Guoqiang Chen, Bing Su, Ying Huang

**Affiliations:** 1 Key Laboratory of Cell Differentiation and Apoptosis of National Ministry of Education, Department of Pathophysiology, Shanghai Jiaotong University School of Medicine, Shanghai, 200025, China; 2 Shanghai Institute of Immunology, Institutes of Medical Sciences, Shanghai Jiao Tong University School of Medicine, Shanghai, 200025, China; University of Parma, ITALY

## Abstract

Sin1 or MAPKAP1 is a key component of mTORC2 signaling complex which is necessary for AKT phosphorylation at the S473 and T450 sites, and also for AKT downstream signaling as well. A number of Sin1 splicing variants have been reported that can produce different Sin1 isoforms due to exon skipping or alternative transcription initiation. In this report, we characterized four Sin1 isoforms, including a novel Sin1 isoform due to alternative 3’ termination of the exon 9a, termed Sin1γ. Sin1γ expression can be detected in multiple adult mouse tissues, and it encodes a C-terminal truncated protein comparing to the full length Sin1β isoform. In contrast to Sin1β, Sin1γ overexpression in Sin1 deficient mouse embryonic fibroblasts has no significant impact on mTORC2 activity or mTORC2 subunits protein level, although it still can interact with mTORC2 components. More interestingly, Sin1γ was detected in a specific cytosolic location with a distinct feature in structure, and its localization was transiently disrupted during cell cycle. Therefore, Sin1γ is a novel Sin1 isoform and may have distinct properties in cell signaling and intracellular localization from other Sin1 isoforms.

## Introduction

The mammalian target of rapamycin (mTOR) is a serine/thronine kinase that is evolutionarily conserved, and it participates in two distinct multiprotein complexes, mTORC1 and mTORC2, which was defined by their sensitivity to rapamycin and their molecular composition [1], [2], [3]. In addition to mTOR, the major conserved components of these two complexes are consisted of mLST8 and raptor for mTORC1, mLST8, rictor and Sin1 for mTORC2, respectively. Over the past decades, studies revealed that the mTOR pathway integrates environmental cues such as nutrients, growth factors and hormones to control cell growth [4]. Growth-promoting signals enhance translation initiation and protein synthesis mainly through upregulation of mTORC1-mediated AGC kinase family members’ phosphorylation [3], including S6K1 hydrophobic motif (HM) site phosphorylation [5], [6]. In contrast to mTORC1, the substrates and biological functions of mTORC2 are less well defined. Phosphorylation of the HM site of Akt requires an intact mTORC2 complex. In addition, mTORC2 is also essential for the growth-independent turn motif (TM)-phosphorylation of Akt and PKCα, which is critical for proper Akt and PKC protein folding [7]. mTORC2 was originally found to regulate actin reorganization but later it was found to control many other important cellular functions such as cell survival and migration [8], [9].

Before Sin1 was known as a unique component of mTORC2 [8], [10], it was originally identified as MEKK2- and Ras-interacting protein which controls MEKK2 dimerization, activation and suppresses Ras signaling. These functions were also confirmed by human Sin1 gene’s orthologous in lower organisms [11],[12]. mSin1 was identified as its mammalian homolog, which could partially compensate the loss of function of ySin-1 in yeast [13]. Interestingly, both mSin1 and ySin1 share a weak homology with AVO1 in S. cerevisiae and RIP3 in D. discoideum[14]. Full length of human Sin1 gene encodes a 522-amino-acid-protein, which could be divided into four domains, N-terminal domain (NTD), conserved region in middle (CRIM), Ras-binding domain (RBD), and pleckstrin homology like domain (PH). Among these domains, NTD is responsible for interaction of Sin1 with other mTORC2 components, without which, functional mTORC2 could not be assembled[15], [16], RBD is known to bind and co-localize with activated H-Ras, and PH domain is important for membrane localization of Sin1 protein. Due to alternative splicing, the Sin1 gene can generate at least five transcript variants that encode four Sin1 isoforms[17]. It was suggested that these isoforms may form distinct mTOR complexes to respond to different upstream signals, but precisely how these isoforms are involved in mTOR signaling remains unclear[18]. It is also unclear whether these Sin1 isoforms may control cellular function independent of the mTOR pathway.

In this study, we examined distribution of Sin1 isoforms and analyzed their roles in mTORC2 signaling. We show that Sin1γ has a unique intracellular localization suggesting that it may have unique function in an either mTOR dependent or independent manner.

## Result

### Characterization of mammalian Sin1 isoforms

**Fig 1:**
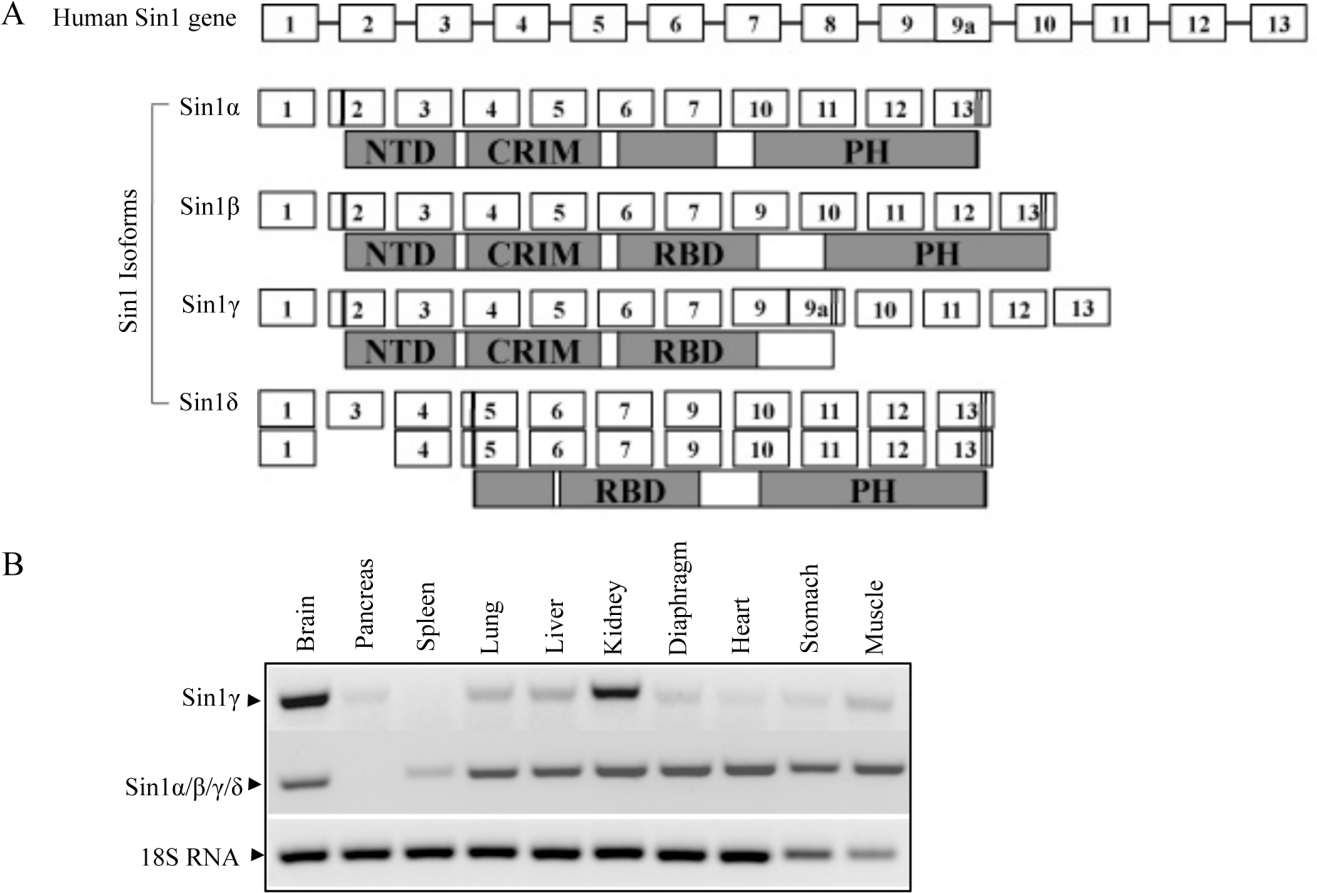
Characterization of Sin1 isoforms. A. Schematic map representing of the human Sin1 gene and transcript variants described in this study. There are five Sin1 transcript variants generating four distinct protein isoforms. Two transcript variants encode for Sin1δ. The green and red solid regions represent start and stop codon respectively. B. Sin1 isoforms are widely express in mouse tissues. Total RNA extracted from mice tissues were analyzed by RT-PCR using either Sin1γ specific primers or primers recognized all four isoforms, 18s RNA were used as loading control. Amplified products were separated by electrophoresis in a 2% agarose gel stained with gel red, demonstrating that all four Sin1 isoforms are widely expressed in mice tissues.

Analyses of the human EST database revealed that human Sin1 gene is composed of at least thirteen exons, corresponding to four putative protein domains, which could give rise to at least five different transcript variants and encode four distinct protein isoforms. As reported previously, Sin1α and Sin1β (accession numbers AY633624 and AY633625) are the longer forms of Sin1 whereas the Sin1γ (accession number AY633626) has a C-terminal truncation. Sequence alignment (Fig 1A) shows that exon8 and 9 are skipped in Sin1α, causing an incomplete RBD in this isoform. Sin1β contains all the exons except exon8 and 9a and encodes the longest isoform or the full length of Sin1 protein. Exon9a with a stop codon is preserved in Sin1γ, which results in the longest cDNA but encodes the shortest form of Sin1 protein with PH domain deletion. A similar C-terminal truncated Sin1, named mSin1.5 was reported before with a slightly different C-terminus[18]. Comparison of the coding sequence of sin1γ with mSin1.5 revealed that they are the most similar transcript variants in terms of their ORFs. Lacking exon 8 and 9, sin1γ mRNA encodes a 372 amino-acid protein while mSin1.5 contains exon 8 that has a premature stop codon generating a slightly small protein product. Interestingly, despite the C-terminal truncation, mSin1.5 was able to assemble the mTORC2 complex and was suggested to facilitate Akt HM-site phosphorylation[18]. It is unclear if Sin1γ, which has a slightly different C-terminus from mSin1.5, could also assemble the mTORC2 complex and to phosphorylate Akt HM-site in vivo. Due to alternative splicing of exon 3, which contains the transcription initiation site, a putative Sin1 isoform, named Sin1δ (accession number AY524429.1 and AL833042.1), may be expressed without NTD domain.

To confirm the expression level of Sin1 isoforms under physiological condition, we designed specific primers to detect the their expression in normal mouse tissues. Using primers that recognized the sequence from the shared exon in all five variants, result indicates a ubiquitously expression of Sin1 mRNA expression. Using Sin1γ special primers, result shows that Sin1γ mRNA also presented in all tissue samples, with the most abundant expression in brain and heart but low in spleen (Fig 1B).

### Sin1 isoforms function in mTOR signaling

**Fig 2:**
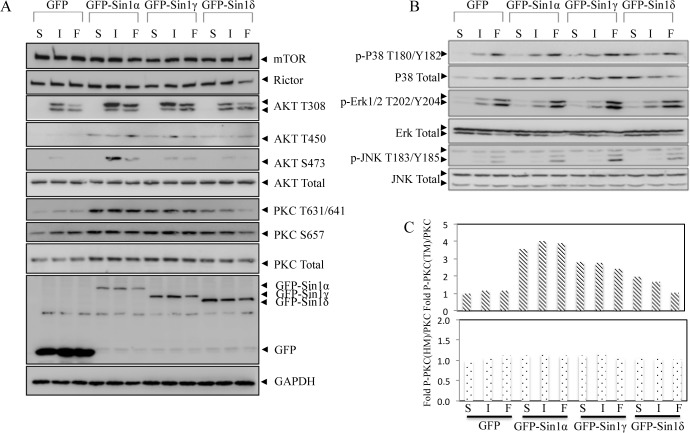
Restoration of Sin1-/- MEFs with GFP-Sin1 expression vectors. A. Sin1 isoforms exert distinct functions in restoration of mTORC2 signaling. Sin1-/- MEF cells transfected with GFP empty vector, GFP-Sin1α, GFP-Sin1β, GFP-Sin1γ, GFP-Sin1δ respectively were grown in starved, or starved then restimulated with insulin or serum for 15min. Total cell lysates were analyzed for indicated proteins by immunoblotting. B. The MAPK pathway is not altered in Sin1 isoform-rescued Sin1-/- MEF cells. Sin1-/- MEF cells transfected with GFP empty vector, GFP-Sin1α, GFP-Sin1β, GFP-Sin1γ, GFP-Sin1δ respectively were grown in starved, or starved then restimulated with insulin or serum for 15min. Total cell lysates were analyzed for indicated proteins by immunoblotting. Single-letter abbreviations for the treatments are as follows: S, starvation for 12hr, I, insulin for 15min after starvation, F, FBS for 15min after starvation. C. The quantifications analysis of the phosphor-PKC band are shown.

To investigate the role of Sin1 isoforms in mTOR signaling, GFP-tagged Sin1 isoforms were transiently expressed in Sin1-/- MEFs separately. The transfected cells were serum starved for 12h followed by restimulation with serum or insulin for another 15min. In GFP empty vector or GFP-Sin1γ, or GFP-Sin1δ expressed Sin1-/- MEF cells, the phosphorylation of Akt HM (AKT S473) site was barely detectable even after serum or insulin treatment (Fig 2A). As reported [8], GFP-Sin1α expression in Sin1-/- MEF cells restored Akt HM phosphorylation after insulin or serum restimulation.

We found that although GFP-Sin1γ or GFP-Sin1δ could not rescue Akt HM site activity, A-Loop phosphorylation of Akt (AKT T308) was almost the same in all groups. These results further support that Sin1 is not required for Akt A-Loop activation. Since TM phosphorylation in Akt (AKT T450) is also Sin1 controlled, growth factor-independent event, we reasoned that some or all Sin1 isoforms could rescue Akt TM phosphorylation activity in Sin1-/- MEF cells. As expected, Akt TM phosphorylation from transfected Sin1-/- MEF cells indicated Sin1α、β、γ but not δ isoforms could rescue TM site activity.

It has been reported that Akt and cPKC displaied very similar sequence at the TM and HM phosphorylation sites [3], and to answer whether a similar regulation was operating in PKC, we also examined the TM (T638) and HM (S657) phosphorylation of PKCα/β in Sin1 isoform reconstituted Sin1-/- MEF cells. In both GFP-Sin1α and GFP-Sin1γ expressed Sin1-/- MEF cells, the TM phosphorylation was presented even in starved condition and this further supports TM site activation is growth factor independent [7]. In contrast, in GFP or GFP-Sin1δ expressed MEF cells, the phosphorylation of TM was almost abolished. On the other hand, there is no obvious difference of HM site phosphorylation among Sin1 isoforms reintroduced MEFs (Fig 2A and 2C). We also compared the rescue capability of GFP-Sin1α and GFP-Sin1β on AKT HM site phosphorylation, the data suggest both of them could resemble mTORC2 function in Sin1 -/- MEF cells (S1 Fig). It is noteworthy that mTOR and rictor protein level were not affected by Sin1 isoform expression (Fig 2A). Taken together, these results indicate GFP-Sin1α and GFP-Sin1β can rescue mTORC2 function while GFP-Sin1γ may partially rescue mTORC2 function but GFP-Sin1δ appeared unable to rescue mTORC2 function in Sin1-/- MEF cells.

Since Sin1 could also interact with MEKK2 and JNK [11],[16], we next examined whether different Sin1 isoforms could affect the MAPK pathways. In Sin1 isoform restored Sin-/- MEF cells, there was no obvious difference in p38, Erk or JNK activation by insulin or serum restimualtion, indicating that Sin1 isoforms may not have different roles in MAPK signaling (Fig 2B).

### Sin1α, β and γ can interact with mTORC2 components.

**Fig 3:**
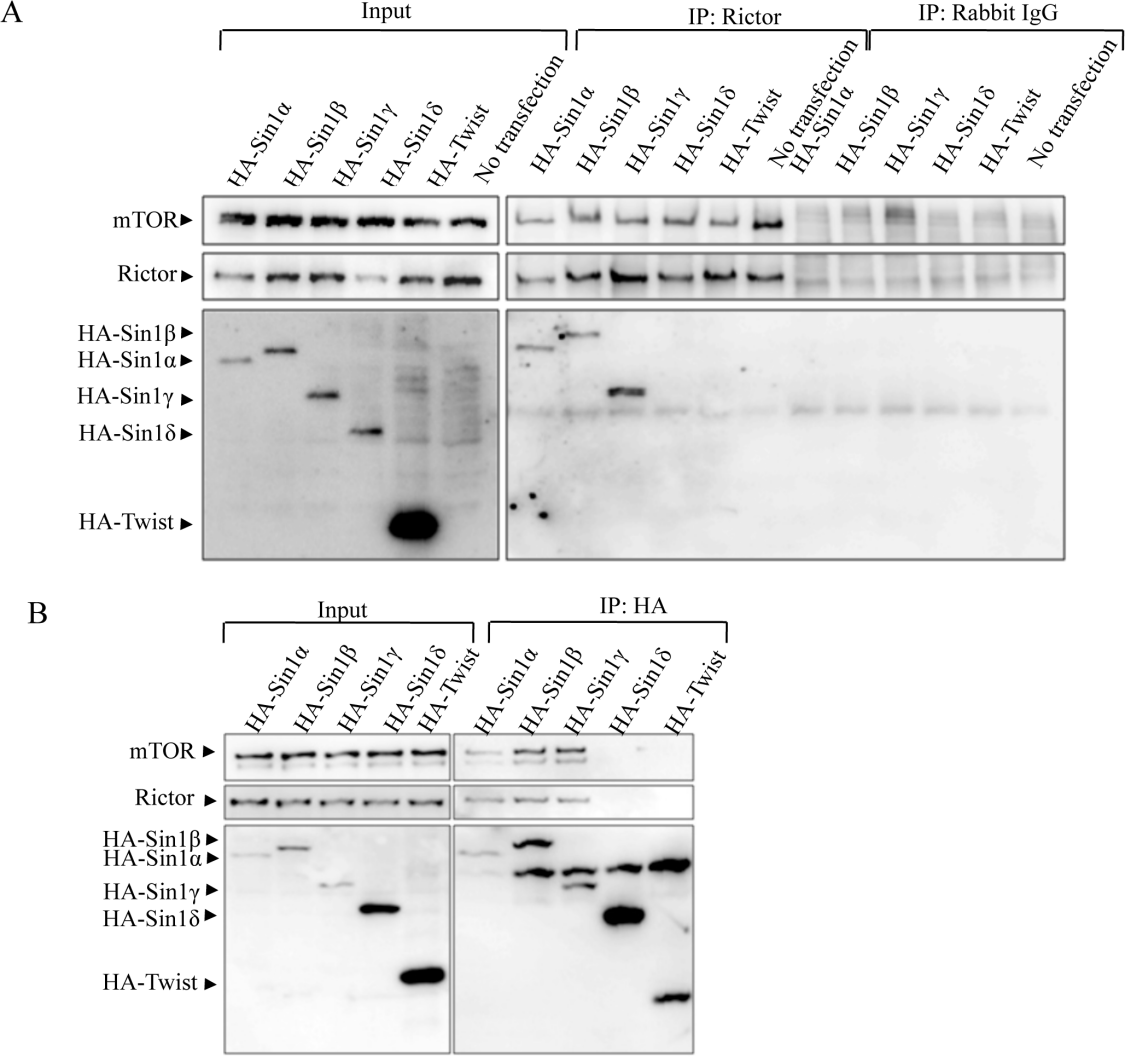
Sin1 isoforms except Sin1δ form mTORC2 complex. A. Rictor pulled down Sin1α, Sin1β, Sin1γ, but not Sin1δ. HEK-293T cells were transiently transfected for 24h with HA-Sin1 isoform plasmids. Cell lysates (left-hand side) and rictor immunoprecipitates (right-hand side) were analyzed for mTOR, rictor and HA by western blotting. B. Sin1α, Sin1β, Sin1γ, but not Sin1δ could pull down rictor and mTOR. HEK-293T cells were transiently transfected for 24h with HA-Sin1 isoform plasmid respectively. Cell lysates (left-hand side) and HA immunoprecipitates (right-hand side) were analyzed for mTOR, rictor and HA by western blotting. All experiments were repeated for three times with the same results.

To determine the interaction of Sin1 isoforms with other mTORC2 components, we transiently expressed HA-tagged Sin1 isoforms in 293T cells (Fig 3A). We immunoprecipitated the HA-tagged Sin1 isoforms and examined its associated mTORC2 components by western blot. Except HA-Sin1δ, HA-Sin1α, β and γ isoforms were able to pull down the endogenous rictor and mTOR (Fig 3B). As a control, a non-related control protein, HA-tagged twist, could not pull down rictor or mTOR. We also performed immunoprecipitation experiment using rictor antibody, and found that rictor could pull down Sin1α, β and γ but not Sin1δ (Fig 3A). These data were consistent with previously published studies showing Sin1α and β were able to assemble the mTORC2 complex[18]. Our data also showed that Sin1γ was able to form functional mTORC2 complex. Again, these data were consistent with previous studies showing that the N-terminal region of Sin1 from 1–93 amino acid is essential for mTORC2 complex assemble [15]. In our result, we also found that Sin1δ was unable to form the core mTORC2 complex (Sin1-mTOR-Rictor). These data partially answered the question why Sin1α, β and γ but not Sin1δ may rescue mTORC2 function.

### Localization of Sin1 isoforms

**Fig 4:**
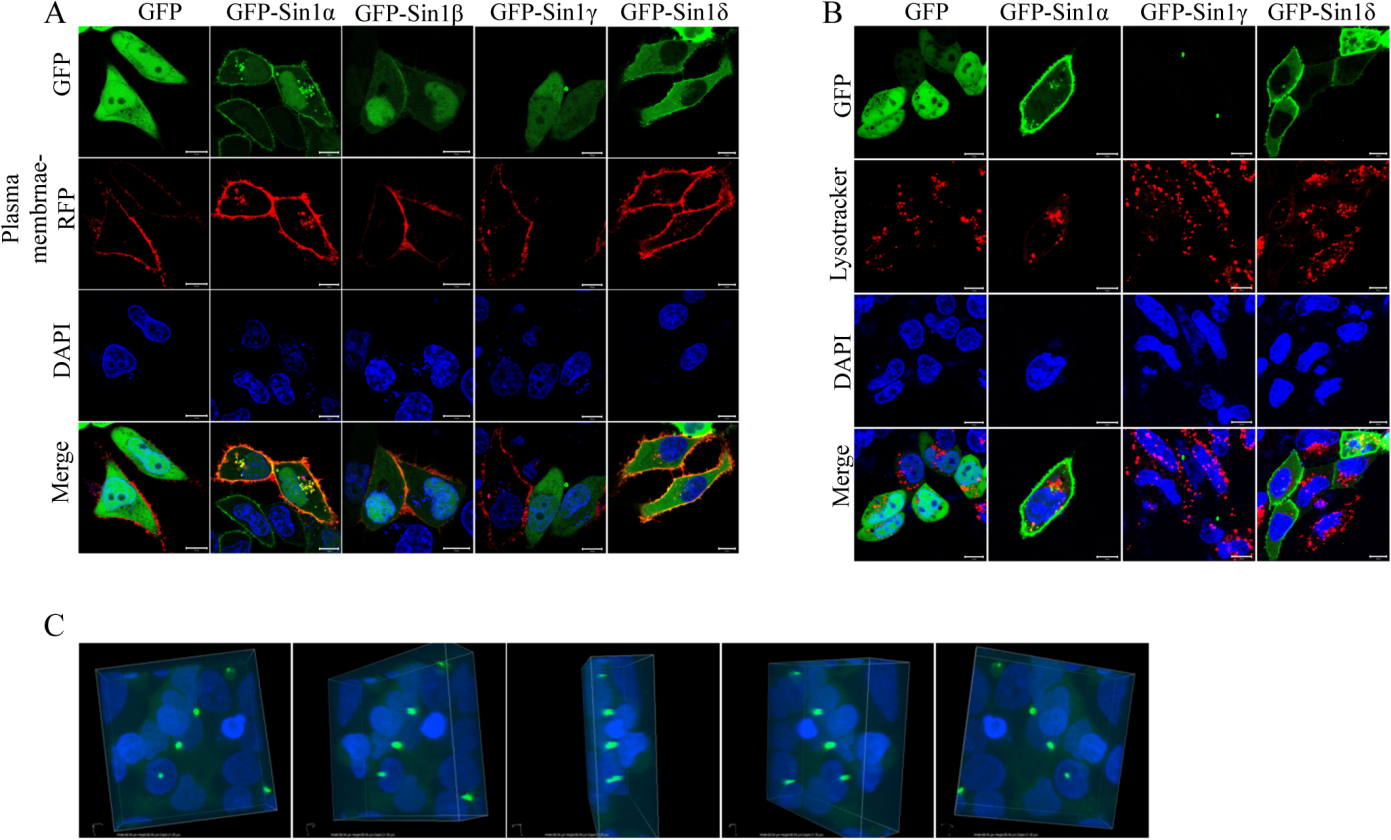
Characterization of localization of Sin1 isoforms. HeLa cell transiently transfected with a plasmid, which express each GFP-tagged Sin1 isoform, were analyzed by confocal microscope and costained with plasma membrane marker (A) or lysotracker (B), (C) 3D structure of GFP-Sin1γ. A series of rotated images demonstrating the cylinder-like structure beside the nuclear. Nuclear was stained by DAPI. These experiments were performed three times and representative images are shown. Scale bars: 10 μm (A, B). All experiments were repeated for three times with the same results.

We speculated that the distinct distributions of Sin1 isoforms could affect their biological function, and investigated the subcellular localization of Sin1 isoforms. We transfected GFP-tagged Sin1 isoforms into Hela cells. Confocal microscopic analyses showed a clear plasma membrane localization of GFP-Sin1α and GFP-Sin1β, and both of them could also be found in the nucleus. Although GFP-Sin1δ was unable to rescue mTORC2 function in Sin1-/- MEF cells, it was found at the plasma membrane and cytosol. To further examine its cellular distribution, we co-stained GFP-Sin1 isoforms transfected HeLa cells with plasma membrane marker conjugated with RFP tag (Fig 4A). The plasma membrane-RFP marker co-localized with all the Sin1 isoforms except GFP-Sin1γ. This result confirmed GFP-Sin1α, GFP-Sin1β and GFP-Sin1δ could localize at the plasma membrane. However, we could not observe colocalization of Sin1 isoforms with ER marker (S2 Fig). Interestingly, in addition to a dispersed distribution in cytoplasm and nucleus, GFP-Sin1γ was found in a unique hollow circular structure outside of the nucleus and only one structure could be found in one cell (Fig 4A and Fig 4B). To further determine the spatial organization of this structure, GFP-Sin1γ was imaged and analyzed by using Zeiss software to restructure at 3D level. When the 3D image of GFP-Sin1γ was rotated, a continuous signal was visible beside the nuclear. This localization pattern indicated GFP-Sin1γ forms a cylinder structure extending outside of the nucleus (Fig 4C).

### Sin1γ colocalizes with basal body

**Fig 5:**
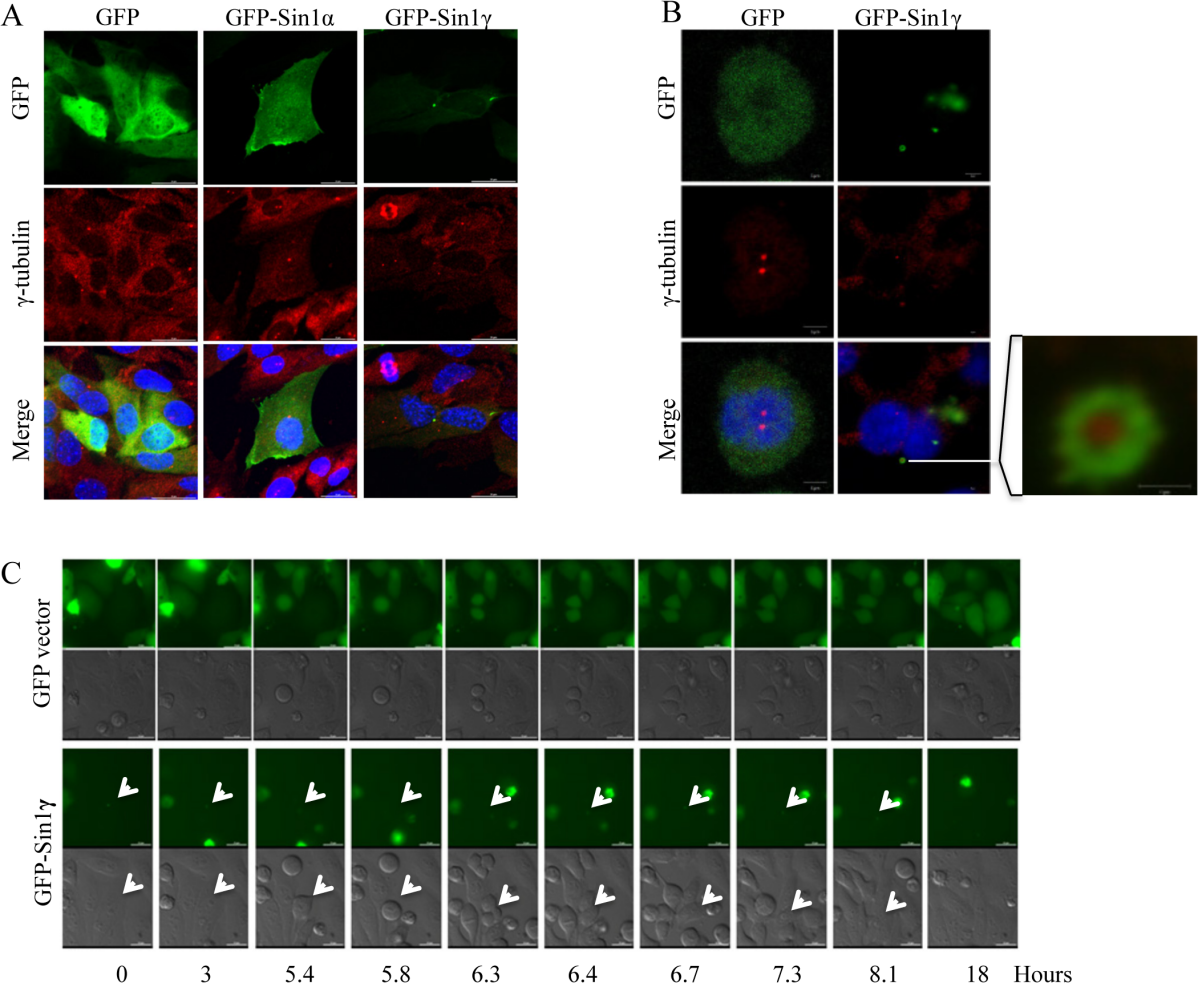
Sin1γ colocalizes with γ-tubulin. A. Sin1-/- MEF cells were transiently transfected with indicated GFP-tagged expression plasmid, the slides were fixed, stained with anti-γ-tubulin/Texas red-labelled anti-mouse secondary antibody and analyzed by confocal microscopy after 24h transfection. B. Hela cells were transiently transfected with indicated GFP-tagged expression plasmid, the slides were fixed, stained with anti-γ-tubulin/Texas red-labelled anti-mouse secondary antibody and analyzed by confocal microscopy after 24h transction. Magnification of colocalization between GFP- Sin1γ and γ-tubulin was shown on the right panel. C. Sin1γ expression fluctuates during cell cycle. HeLa cells transfected with GFP empty vector or GFP-Sin1γ for 24h were subjected to time-lapse microscopy observation. Both GFP and DIC channels were shown for cells transfected with each expression plamids. Scale bars: 25 μm (A and C), 5 μm (B). All experiments were repeated for three times with the same results.

To gain more insight of the cellular localization of Sin1 isoforms, we also utilized lysotracker to determine if some of the GFP-Sin1 isoforms may associate with lysosome as it was proposed that mTOR controls some lysosomal functions in an amino acid dependent manner. The data clearly indicated GFP-Sin1γ could not colocalize with lysotracker, while a small portion of GFP-Sin1α expressed cells appeared to have colocalized with lysosome (Fig 4B). Interestingly, we found that most of the cells having the GFP-Sin1γ cylinder structure were in interphase (data not show), this suggested that special structure might be localized at centrosome. To test this possibility, we stained centrosome marker γ-tubulin and determined if γ-tubulin colocalize with GFP-Sin1γ in transfected Sin1-/- MEF cells[19]. Consistent with the results in HeLa cells, GFP signal diffused distributed within the cell, GFP-Sin1α signal could be found at the plasma membrane and cytoplasm, with no overlap with γ-tubulin, while GFP-Sin1γ formed a cylinder structure and appeared to colocalize with γ-tubulin (Fig 5A). Examination at a higher magnification under confocal microscope further revealed their overlapping pattern, basing on that GFP-Sin1γ appeared to surrounded γ-tubulin (Fig 5B). This result suggested that GFP-Sin1γ might uniquely colocalize with γ-tubulin and contribute to centrosome assembly and function.

**Fig 6:**
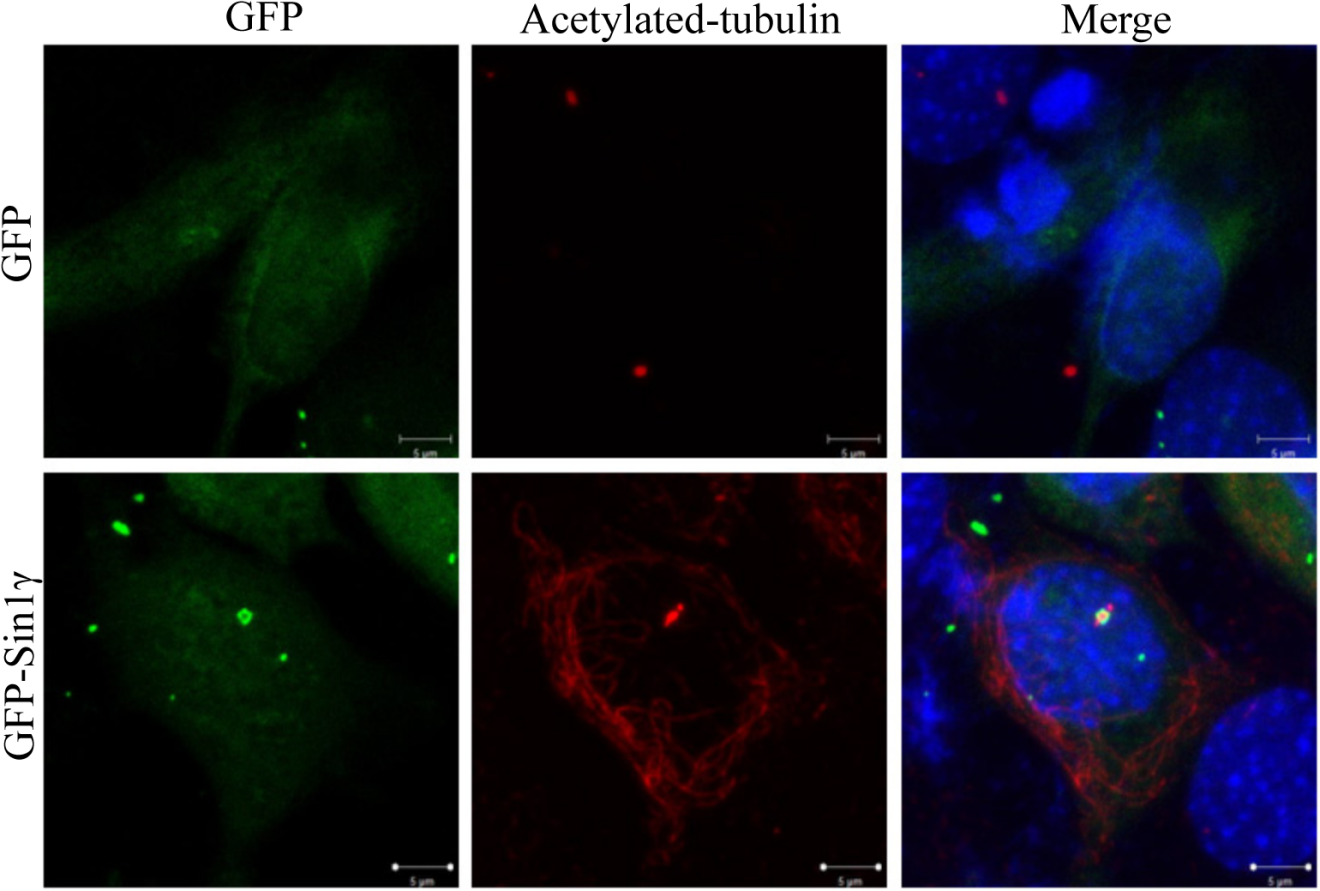
Sin1γ colocalizes with basal body. Sin1-/- MEF cells were transiently transfected with GFP empty vector or GFP-Sin1γ, the slides were fixed, stained with anti-acetylated-tubulin/Texas red-labelled anti-rabbit secondary antibody and analyzed by confocal microscopy after 24h transfection. Scale bars: 25 μm (A), 2.5 μm (B and C). All experiments were repeated for three times with the same results.

If GFP-Sin1γ is indeed a part of centrosome, it may generate two cylinder structures and distribute evenly into two daughter cells during mitosis [20]. To examine this possibility, HeLa cells were transfected with GFP empty vector or GFP-Sin1γ for 12 hours and subjected to time-lapse microscope observation for 18hours. HeLa cells expressing control GFP alone underwent mitosis and GFP signal could be detected during the whole cell-cycle time frame. To our surprise, the GFP positive cylinder structure in GFP-Sin1γ expressing HeLa cells could be clearly seen before cells entering mitosis. However, when the cell was getting round up, this structure disappeared but re-appeared after mitosis (Fig 5C). This finding suggested that GFP-Sin1γ might not function as microtubule organization center (MTOC) during cell cycle. Since γ-tubulin is also a structure base of basal body [21], we examined if the cylinder structure of GFP-Sin1γ could co-stain with basal body marker acetylated-tubulin [22], [23]. Indeed, GFP-Sin1γ formed cylinder structure surrounding acetylated-tubulin (Fig 6). This data suggested that GFP-Sin1γ was associated with basal body and its role in basal body function was worth to be further explored.

### Knockdown Sin1 protein inhibits cilia formation

**Fig 7:**
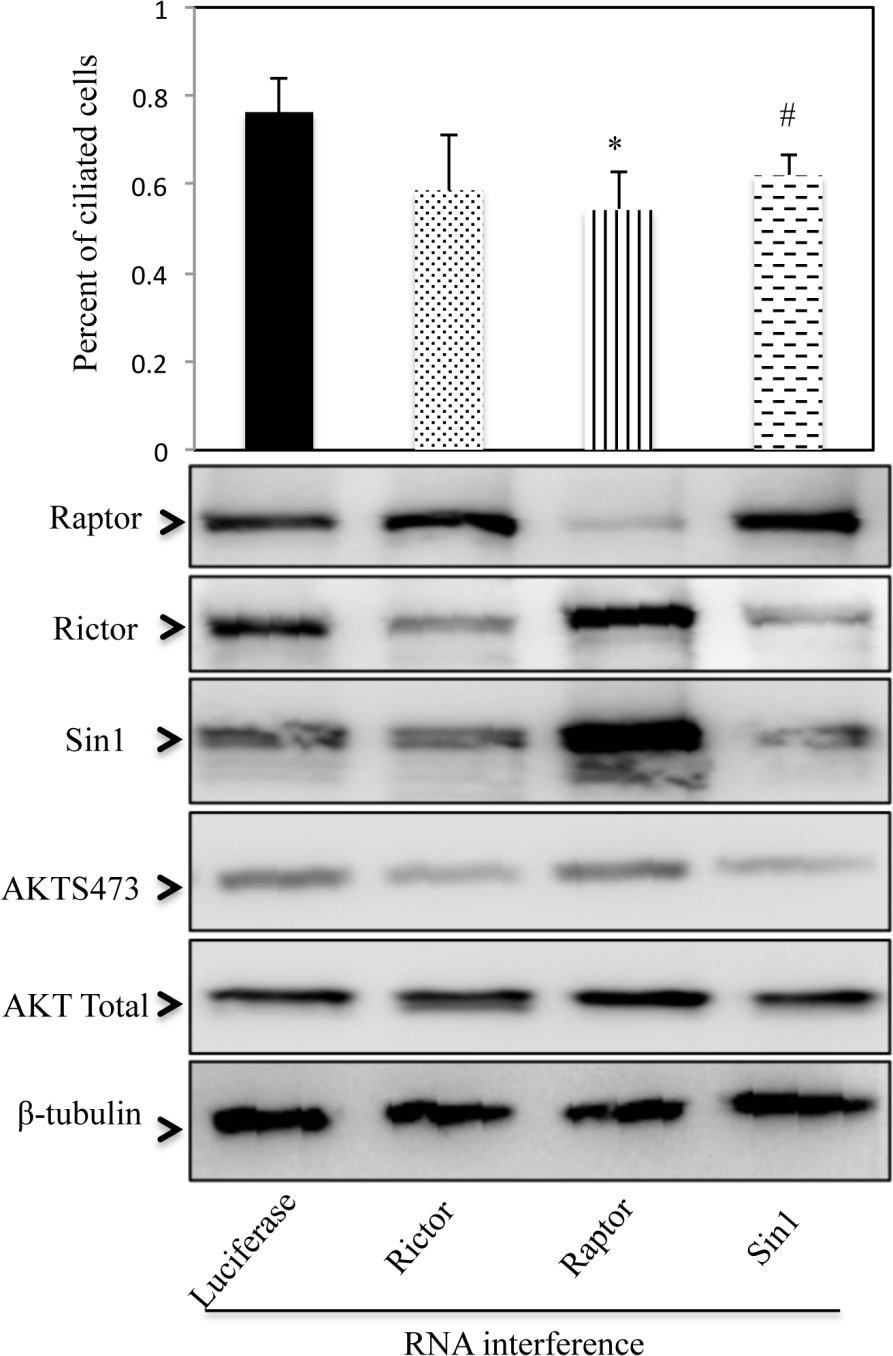
Knockdown Sin1 protein inhibits cilia formation. A. The percentage of RPE1 cells with cilia were determined using acetylated-tubulin staining. Average data obtained from two to three different views are shown. Approximately 300 cells for each siRNA transfection were scored each time. Error bar represent SD. *p<0.01 compared with luciferase, #p<0.05 compared with luciferase. B. siRNA knockdown efficiency from (A) were detected using western blot.

Primary cilium is composed of basal body, we next examined whether decreased Sin1 protein level will affect cilia formation. Rictor, Raptor or Sin1 specific SiRNAs were transfected into RPE1 cells respectively, the percentage of ciliated cells was counted (Fig 7). Our result indicated the incidence of serum-starvation-induced ciliogenesis in Raptor knockdown cells dropped by 22% when compared with cells transfected with luciferase SiRNA, which was consistent with previously reported that mTORC1 components were related to cilia formation [24]. The percentage of ciliated cells in Sin1 SiRNA expressed group were also statically decreased as we expected, wherase the silencing of Rictor had no such effect. These results demonstrated the involvement of Sin1 gene in the cilia formation.

## Discussion

Current understanding of mTORC2 function and regulation is still lagged behind that of mTORC1. Sin1 is an essential mTORC2 component required for the complex integrity and substrate specificity. However, due to alternative splicing, a single Sin1 gene generates multiple Sin1 isoforms, but their contribution to mTORC2 function as well as other cellular function remains largely unclear. At least three Sin1 isoforms could assemble into mTORC2 complex but their precisely in vivo roles and regulation at the molecular levels are still poorly understood.

To better understand Sin1 function, in this study we characterized four Sin1 isoforms, Sin1α, Sin1β, Sin1γ and Sin1δ, in terms of their roles in mTORC2 signaling, their capability in forming mTORC2 complexes, and their intracellular localization. We first compared their abilities to restore mTORC2 activity in Sin1-/- MEF cells. Ectopic expressed Sin1α almost fully rescued AKT HM site phosphorylation in Sin1-/- MEF cells, while Sin1γ could only weakly restore AKT HM site phosphorylation but Sin1δ could not restore at all. Although this functional difference could be due to many reasons, one possibility is these Sin1 isoforms have different cellular distributions.

Sin1β is the longest isoform among the four Sin1 isoforms. As shown previously, it could interact with rictor and mTOR to form intact mTORC2 complex, and rescue AKT HM phosphorylation in Sin1-/- MEF cells. Sin1α is the second longest isoform with a truncated RBD domain with similar ability as Sin1β to form mTORC2 complex and restore Akt HM phosphorylation. Sin1δ lacks NTD, which could not rescue mTORC2 function in Sin1-/- MEF cells. Using Co-IP assay, we also found that its overexpression in 293T cells was not associated with mTORC2 complex. This result was not surprising since in the Sin1-/- MEF cells, this isoform may exist in theory.

Sin1γ is a novel Sin1 isoform which has not been reported before. It is the most similar one to mSin1.5 which was reported before, except that the C-terminus is different due to the alternative use of the exon 8 and 9. Missing the C-terminal PH-like domain could impair its ability to associate with plasma membrane, a step that is believed to be required for mTORC2 to phosphorylate Akt HM site. Indeed, expressing this isoform in Sin1-/- MEF cells only weakly restored Akt HM site phosphorylation as compared to Sin1α or Sin1β. Interestingly, this isoform still interacts with rictor and is able to assemble the mTORC2 complex similar to mSin1.5 as reported previously [18]. However, in contrast to the previous conclusion that mSin1.5 is able to assemble functional mTORC2 to phosphorylate Akt HM site, our data suggested that it could not do so in vivo.

In addition to Akt, we also examined PKCα TM and HM sites phosphorylation. Our data show that Sin1α overexpression could restore the PKCα TM phosphorylation. However, there is no obvious difference of PKCα HM phosphorylation level among Sin1 KO MEF cells and Sin1 isoform transfected MEF cells. It is possible that PKC HM site phosphorylation is not mTORC2 dependent [7].

As Sin1 was originally identified to bind serine/threonine kinases MEKK2 and SAPK/JNK in the MAPK modules, we also examined if Sin1 isoforms may differentially affect MAPK activation. However, expressing different Sin1 isoforms in Sin1-/- MEF cells had no major effect on serum or insulin induced MAPK activation, indicating that Sin1 isoforms may not participate in growth factor related MAPK activation.

Using confocal microscope and time lapse imaging, we examined the cellular localization of Sin1 isoforms. PH-like domain is responsible for Sin1 plasma membrane localization, thus Sin1α, Sin1β and Sin1δ could be detected at plasma membrane and co-stained with plasma membrane marker. These results were consistent with previous studies reported by Wayne Schroder et al [12]. More interestingly, lacking of N-terminal domain, Sin1δ could not be observed in the nuclear, comparing to Sin1α, Sin1β and Sin1γ, indicating that there might be NLS within NTD domian. The presence of NLS might endow Sin1 isoforms some mTOR-independent functions. However, Sin1 isoforms appeared not colocalizing with ER marker. The reason for this discrepancy between our work and that of Sarbassov’s [25]finding was unclear. It could be due to different cells has been used in these different studies.

A key finding in our study was that a novel Sin1 isoform, Sin1γ, shown a specific cellular localization at the basal body, in addition to a diffused cellular distribution. Basal body is an organelle formed from a centriole and has been found as the initiator of eukaryotic cilium. Interestingly, the cylinder structure of Sin1γ was found to surround the centriole marker γ-tubulin. In addition, this unique structure was cell cycle dependent, which disappeared during mitosis and re-appeared at interphase. Furthermore, it colocalized with acetylated-tubulin, a marker for basal body. We also performed Co-IP to confirm the interaction of Sin1γ and acetylated-tubulin. Unfortunately, the result showed Sin1γ could not pull down acetylated tubulin (S3 Fig). Since basal body is a structure component of cilia, we also further analyzed Sin1’s role in cilia formation. Due to the difficulty of designing Sin1γ specific SiRNA, we compared the effects of silencing Raptor, Rictor or Sin1 individually on cilia formation. No surprisingly, inhibited Raptor expression attenuated cilia formation in RPE1 cells. Sin1 SiRNA expression also deceased percentage of ciliated cells while Rictor SiRNA had no such effect. Although, we did not observe the change of cilia length in those SiRNA expressed cell [26]. Finally, Sin1γ was highly expressed in kidney, where the primary cilia played a pivotal role in pathogenesis of polycystic kidney disease. It would be interesting to determine if Sin1γ may paly a role in kidney disease.

It has been reported Sin1 isoforms defined three distinct mTORC2, mSin1.5-containing mTORC2 could not respond to insulin induced AKT HM-site phoshorylation. Sequence alignment revealed mSin1.5 is the most similar isoform to Sin1γ. Although our data indicated that Sin1γ exerts minor mTORC2 activity upon serum or insulin stimuli which could be due to the tail sequence difference. Owing to similarity among Sin1 isoforms, we could not design isoform specific SiRNA to confirm the individual role in mediating mTORC2 substrates activation. An alternative approach that worthy trying is to detect their tissue expression pattern.

In summary, our data demonstrated that Sin1 isoforms (Sin1α, sin1β, sin1γ and sin1δ) might have different function basing on their protein structure and distinctive cellular distribution. Among them, Sin1γ is a novel isoform with unique basal body co-localization and with decreased mTORC2 activity. Our work shed light on mTORC2 functions and helped to add insights on the understanding of mTORC2 components.

## Materials and Methods

### Cell culture, stimulation, transient transfection and infection

MEFs were originated from embryos at embryoic day10 and maintained in DMEM with 10% FBS as described [8]. Immortalized MEFs were generated spontaneously. HEK293T, HeLa and immortalized Sin1-/- MEF cells were cultured in Dulbecco’s modified Eagle’s medium (DMEM) (Hyclone, Beijing), hTERT-RPE1 cell in a 1:1 mix of DMEM and F12 (Invitrogen) and 0.01 mg/ml hygromycinB, both supplemented with 10% heat-inactivated fetal calf serum (FBS, Gibco BRL, Gaithersburg, ML), penicillin (100 IU/ml) and streptomycin (100 μg/ml) in a humidified incubator at 37°C and 5% CO2/95% air. Cells were FBS starved for 12h, then stimulated with 1ug/ml insulin or 10% FBS. Plasmids were transfected using lipofectimine2000 (Invitrogen). After 24hr of transient transfection, GFP-Sin1 isoform expressed HeLa cell was incubated with Cell light plasma-membrane baculovirus (Invitrogen molecular probe) for another 16hr or incubated with Lysotracker for 15min followed by microscope examination.

### Plasmids and cloning

HA-tagged Sin1α, Sin1β, Sin1γ and Sin1δ in pMigw vectors have been previously described [8]. GFP-tagged sin1α, sin1β, sin1γ and sin1δ were constructed follow standard cloning procedure. Briefly, Sin1 isoforms were amplified from pMigw-Sin1 plasmids and inserted into EGFP-C1 plasmid with HindIII and SalI sites. The authenticity of all plasmids was confirmed by sequencing.

### RNAi interference

Small interfering RNAs (siRNAs) of human Sin1, Rictor and Raptor were synthesized from genepharma, the corresponding sequences are available upon request. Briefly, cells seeded at 70% confluency in an antibiotic-free culture medium were transfected with siRNA duplexes at a final concentration of 100pM for 48hrs. The capability of silencing related genes for these sequences were confirmed by western blot.

### Tissue preparation and RT-PCR

Animals were euthanized by CO_2_ for collection of tissues. Animal handling was approved by the committee for Humane Treatment of Animals at Shanghai Jiaotong University School of Medicine. C57 WT mice originated frozen tissues were ground with a pestle and mortar in a liquid nitrogen bath. The resulting powders were dissolved in TRIzol reagent (Invitrogen) for RNA. Reverse transcription was performed with 1ug of total RNA. The primer pairs were used in RT-PCR as followed:

Mouse Sin1γF1 5’-ggcagtgaaaagaagaaaagga-3’

Mouse Sin1γR1 5’-agcatgtttgcaacagacaaag-3’

Mouse Sin1α/β F3 5’-actccgcaaagagagacaaaac-3’

Mouse Sin1α/β R3 5’-actcctgggctgattgtttaga-3’

Mouse 18sRNA F 5'-aggccctgtaattggaatgagtc-3'

Mouse 18sRNA R 5'-gctcccaagatccaactacgag-3'

Traditional PCR was performed with cDNA, equivalent to 0.04ug of RNA, 2X taq PCR mixture (LifeFeng), 10pmol of each PCR primer. The PCR reactions were amplified in Bio-rad C1000TM Thermal cycler with the following cycling protocol: 94°C denaturation for 5min, 94°C, 30s– 60°C,30s–72°C, 30s, for 35 cycles, after cycling was completed there was a final extension of 10min at 72°C. The PCR reaction products were running in 2% agrose gel and visualized by Gel-red fluorescent dye.

### Cell lysis, immunoprecipitation and immunoblotting

Cells were washed twice with ice-cold PBS and harvested in 1xCell Lysis Buffer (Cell signaling) with proteinase inhibitor cocktail. Protein concentration was determined using bradford protein assay kit (Shenggong, shanghai) and 30μg of each samples were separated on 8–12% SDS-PAGE and transferred onto a nitrocellulose blot (Amersham Biosciences). Immunoprecipitation of mTOR complexes were performed as previously described with minor modification [8]. Briefly, total HEK-293T cell extracts were lysed with CHAPS lysis buffer (40 mM Hepes pH 7.5, 120 mM NaCl, 0.3% CHAPS, 1 mM EDTA, 10 mM pyrophosphate, 10 mM glycerophosphate, 50 mM NaF, 0.5 mM orthovanadate) in total 500ul volume, cleared by 20min centrifugation at 12,000rpm, and then incubated with 0.5μg corresponding antibody (Rictor(Bethyl), HA(Sigma)) or rabbit IGg (Santa Cruz), on a rotate at 4°C for overnight, then followed by addition of 15 μl protein G-agarose (Roche) and incubated for an additional 1.5 hours. Immunoprecipitates captured by protein G-agarose were washed 5 times with CHAPS lysis buffer and then boiled at 95°C. Samples were then subjected to immunoblotting analysis. The blot was probed with the indicated primary antibodies (mTOR, Rictor, p-AKT T308, p-AKT T450, p-AKT S473, AKT total, p-PKCαT631/T641, PKCα total, p- FoxO1/3a T24, FoxO1/3a total, p-P38 T180/Y182, P38 total, p-Erk1/2 T202/Y204, Erk total, p-JNK T183/Y185, JNK total, GADPH(Cell signaling), GFP(Sigma), HA(Convance)). Protein signals were detected using HRP-conjugated secondary antibodies (Cell signaling) and enhanced chemiluminescence (ECL) Western blotting detection regents (Pierce).

### Immunofluorescence assays and fluorescent microscopy

For living cells, after 24hr of transient transfection, GFP-Sin1 isoform expressed HeLa cells were incubated with Cell light plasma-membrane baculovirus (Invitrogen molecular probe) for another 16hr or incubated with Lysotracker for 15min followed by microscope examination. To prepare slides, cells were washed in PBS and fixed with 4% formaldehyde for 10 minutes. After permeabilized by 0.3% Triton X-100 in PBS, cells were incubated with 1% bovine serum albumin (BSA), followed by incubating overnight with antibodies to γ-tubulin (Sigma-Aldrich, St Louis, MO) or acetylated-tubulin (Abcam). Then, cells were stained with appropriate second antibodies conjugated with Alex Fluor-555 (Invitrogen) for 1 hour. Cellular DNA were finally stained with 4’,6’-diamidino-2-phenylindole (DAPI, Molecular Probe, Eugene, OR). Fluorescence signals were detected on a Zeiss 710 laser scanning confocal microscope. Three-dimensional images were reconstructed from the contours defined at each confocal plane. For time-lapse video imaging, HeLa cells seeded onto chamber slides were transfected with various expressing constructs for 24hr. The transfected cells were then cultured in a humidified chamber at 37°C in Dulbecco’s modified Eagle’s medium (DMEM) supplemented with 10% heat-inactivated fetal calf serum (FBS, Gibco BRL, Gaithersburg, ML), penicillin (100 IU/ml) and streptomycin (100 μg/ml). Cells ectopically expressing transfected plasmids were subjected to time-lapse video imaging on Nikon Ti-E microscope.

## Supporting Information

S1 Fig Both Sin1α and Sin1β can restore AKT HM site activity. Sin1-/- MEF cells transfected with GFP empty vector, GFP-Sin1α or GFP-Sin1β, respectively were grown in starved, or starved then restimulated with insulin or serum for 15min. Total cell lysates were analyzed for indicated proteins by immunoblotting. All experiments were repeated for three times with the same results.
(TIF)

S2 Fig Sin1 isoform could not colocalize with ERtracker. HeLa cell transiently transfected with a plasmid, which express each GFP-tagged Sin1 isoform, were analyzed by confocal microscope and costained with ERtracker. The experiment was repeated at least three times and representative images were shown. Scale bars: 10 μm. All experiments were repeated for three times with the same results.
(TIF)

S3 Fig Sin1 isoform cannot pull down acetylated-tubulin. HEK-293T cells were transiently transfected for 24h with HA-Sin1 isoform plasmid respectively. Cell lysates (left-hand side) and HA immunoprecipitates (right-hand side) were analyzed for mTOR, HA, tubulin and acetylated-tubulin by western blotting. All experiments were repeated for three times with the same results.
(TIF)
