# Supplementary figures and images for: Characterization of Sin1 Isoforms Reveals an mTOR-Dependent and Independent Function of Sin1γ
Source: PLoS One. 2015 Aug 11;10(8):e0135017. doi: 10.1371/journal.pone.0135017 (PMC4532406; doi:10.1371/journal.pone.0135017)

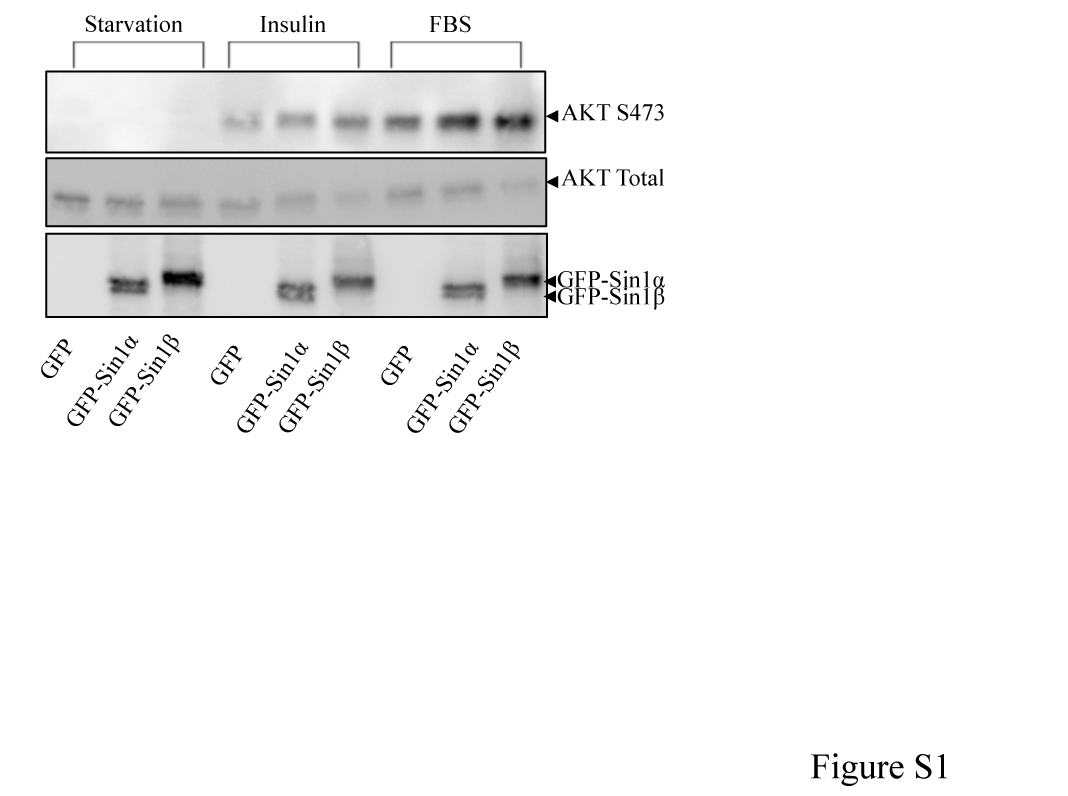

Supplement: S1 Fig — Sin1-/- MEF cells transfected with GFP empty vector, GFP-Sin1α or GFP-Sin1β, respectively were grown in starved, or starved then restimulated with insulin or serum for 15min. Total cell lysates were analyzed for indicated proteins by immunoblotting. All experiments were repeated for three times with the same results. (TIF) [file pone.0135017.s001.tif]

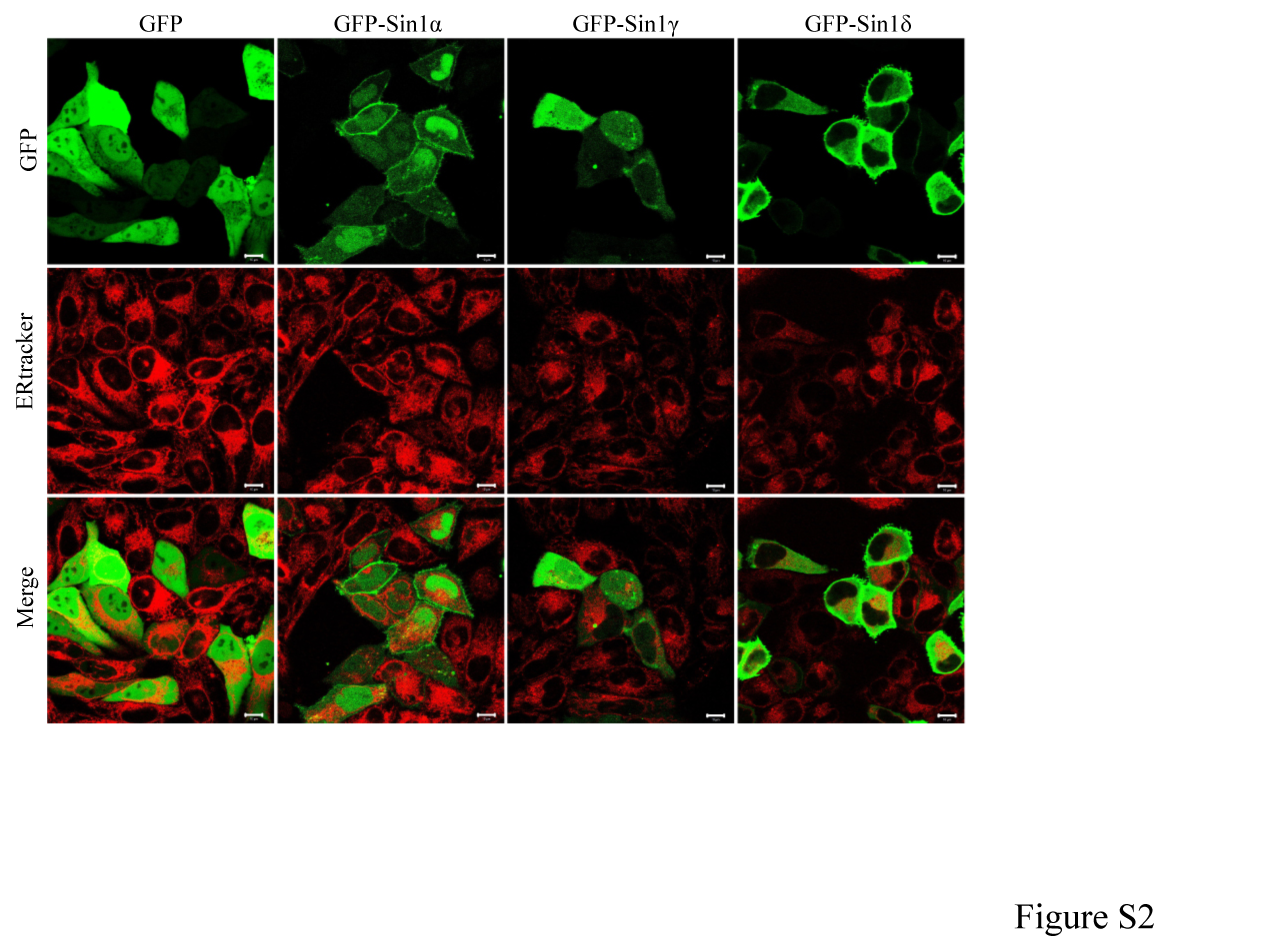

Supplement: S2 Fig — HeLa cell transiently transfected with a plasmid, which express each GFP-tagged Sin1 isoform, were analyzed by confocal microscope and costained with ERtracker. The experiment was repeated at least three times and representative images were shown. Scale bars: 10 μm. All experiments were repeated for three times with the same results. (TIF) [file pone.0135017.s002.tif]

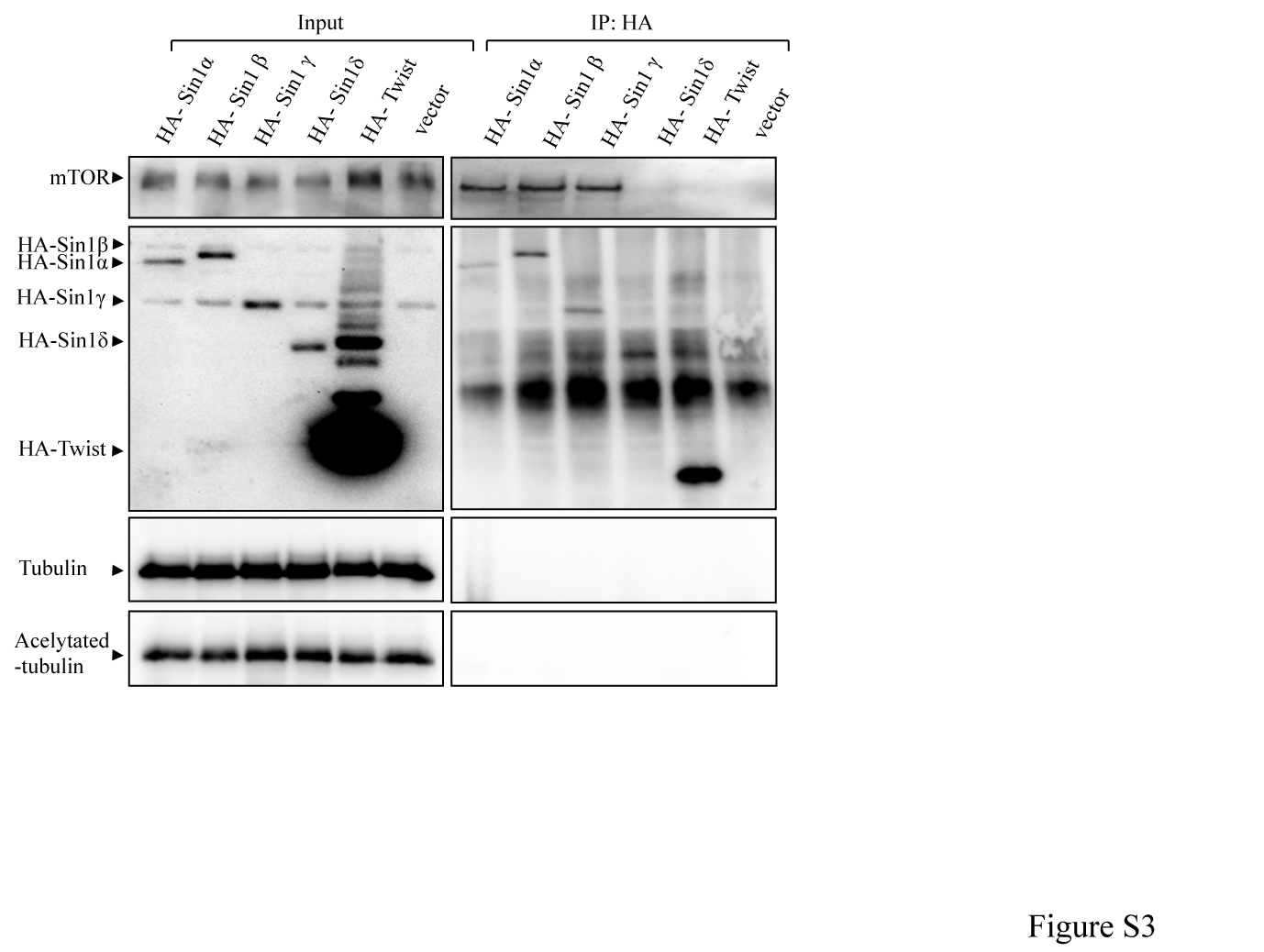

Supplement: S3 Fig — HEK-293T cells were transiently transfected for 24h with HA-Sin1 isoform plasmid respectively. Cell lysates (left-hand side) and HA immunoprecipitates (right-hand side) were analyzed for mTOR, HA, tubulin and acetylated-tubulin by western blotting. All experiments were repeated for three times with the same results. (TIF) [file pone.0135017.s003.tif]
